# Prevalence of WHO Transmitted Drug Resistance Mutations by Deep Sequencing in Antiretroviral-Naïve Subjects in Hunan Province, China

**DOI:** 10.1371/journal.pone.0098740

**Published:** 2014-06-04

**Authors:** Zou Xiaobai, Chen Xi, Hongping Tian, Ann B. Williams, Honghong Wang, Jianmei He, Jun Zhen, Jennifer Chiarella, Lisebeth A. Blake, Gregory Turenchalk, Michael J. Kozal

**Affiliations:** 1 Hunan Provincial Center for Disease Control and Prevention, Changsha, Hunan Province, China; 2 Yale-China Association, New Haven, Connecticut, United States of America; 3 UCLA School of Nursing, Los Angeles, California, United States of America; 4 Central South University, Hunan Province, China; 5 Yale School of Medicine, New Haven, Connecticut, United States of America; 6 454 Life Science, Branford, Connecticut, United States of America; Centro de Biología Molecular Severo Ochoa (CSIC-UAM), Spain

## Abstract

**Background:**

There are few data on the prevalence of WHO transmitted drug resistance mutations (TDRs) that could affect treatment responses to first line antiretroviral therapy (ART) in Hunan Province, China.

**Objective:**

Determine the prevalence of WHO NRTI/NNRTI/PI TDRs in ART-naïve subjects in Hunan Province by deep sequencing.

**Methods:**

ART-naïve subjects diagnosed in Hunan between 2010–2011 were evaluated by deep sequencing for low-frequency HIV variants possessing WHO TDRs to 1% levels. Mutations were scored using the HIVdb.stanford.edu algorithm to infer drug susceptibility.

**Results:**

Deep sequencing was performed on samples from 90 ART-naïve subjects; 83.3% were AE subtype. All subjects had advanced disease (average CD4 count 134 cells/mm^3^). Overall 25.6%(23/90) of subjects had HIV with major WHO NRTI/NNRTI TDRs by deep sequencing at a variant frequency level ≥1%; 16.7%(15/90) had NRTI TDR and 12.2%(11/90) had a major NNRTI TDR. The majority of NRTI/NNRTI mutations were identified at variant levels <5%. Mutations were analyzed by HIVdb.stanford.edu and 7.8% of subjects had variants with high-level nevirapine resistance; 4.4% had high-level NRTI resistance. Deep sequencing identified 24(27.6%) subjects with variants possessing either a PI TDR or hivdb.stanford.edu PI mutation (algorithm value≥15). 17(19.5%) had PI TDRs at levels >1%.

**Conclusions:**

ART-naïve subjects from Hunan Province China infected predominantly with subtype AE frequently possessed HIV variants with WHO NRTI/NNRTI TDRs by deep sequencing that would affect the first line ART used in the region. Specific mutations conferring nevirapine high-level resistance were identified in 7.8% of subjects. The majority of TDRs detected were at variant levels <5% likely due to subjects having advanced chronic disease at the time of testing. PI TDRs were identified frequently, but were found in isolation and at low variant frequency. As PI/r use is infrequent in Hunan, the existence of PI mutations likely represent AE subtype natural polymorphism at low variant level frequency.

## Introduction

Hunan is the 11th largest province of China, situated in the southeast region of the country. Commercial sex work and injection drug use are highly prevalent; and the HIV/AIDS epidemic has expanded rapidly. Through the end of 2012 more than 16,000 HIV-infected cases have been reported in Hunan Province [1]–[4]; Hunan HIV epidemiology and treatment database system. More than 6,600 HIV-infected patients are in care and have received free antiretroviral therapy (ART) which is supported by the government. Currently, 5,133 patients remain on ART (Hunan HIV epidemiology and treatment database system). Prior to 2012 in Hunan Province, the first line ART consisted of triple therapy with 2 nucleos(t)ide reverse transcriptase inhibitors (NRTIs) with a non-nucleoside reverse transcriptase inhibitor (NNRTI) - available first line agents were: stavudine (d4T), lamivudine (3TC), zidovudine (AZT), tenofovir (TDF) and nevirapine (NVP). Boosted protease inhibitors (PI/r) have been used infrequently in Hunan. Lopinavir/ritonavir (LPV/r) could be used in second line regimens; however, there has not been wide scale use of this agent in Hunan Province as of yet. Routine HIV viral load (VL) testing is generally performed once a year in patients on ART.

Although ART is very successful in treatment HIV/AIDS, the efficacy of first line regimens can be reduced by the presence of transmitted drug resistance mutations (TDRs). There are few data on the prevalence of World Health Organization (WHO) TDRs that could affect treatment responses to the first line regimen for ART-naive HIV-infected persons in Hunan. HIV subtype AE predominates in Hunan, but data on the prevalence of low frequency HIV variants with NRTI, NNRTI and PI resistance mutations among this subtype are limited. Currently available conventional genotyping sequencing does not detect low-level resistant variants at levels less than 20% of the circulating viral quasispecies [5]. There are reports that low abundance drug resistant HIV variants at levels as low as 1% of the circulating viral quasispecies can be detected in ART-naïve individuals by more sensitive and quantitative genotyping technologies [5]–[20]. Low-frequency NNRTI drug-resistant variants below the 20% level have been strongly associated with virologic failure for subjects initiating NNRTI-based therapy [16]–[20].

In this study, we investigated the prevalence of WHO NRTI and NNRTI TDRs that can affect the treatment responses to first line ART among ART-naïve HIV infected subjects entering care in Hunan Province, China. In addition, we determined the prevalence of WHO TDR and Stanford.HIVdb.edu PI resistance mutations in ART-naïve subjects.

## Materials and Methods

### Ethics Statement

The study was conducted at two clinical sites, in Hengyang City and Changsha City, Hunan Province. At each site, clients receive comprehensive evaluation and ART when indicated. The study was approved by the Human Investigative committees/IRBs at Yale University, New Haven, CT, USA and Central South University, Hunan Province, China and all subjects in the study gave written informed consent.

### Setting and Subjects

Throughout China and in Hunan, the national China CARES program provides clinical evaluation and free medication to HIV-infected individuals. Patients attending the China CARES clinics are poor and primarily rural residents. All individuals presenting for initial anti-retroviral therapy at one of the two largest China CARES sites (Changsha and Hengyang) in Hunan between July 5, 2010 and August 8, 2011 were invited to participate in this descriptive cross sectional study of HIV-RNA quantity and genotype. The two study sites provide care to 35.9% (1065/2970) of provincial China CARES patients.

All subjects were18 years of age or older, understood and spoke Mandarin, and were mentally competent to answer questions in the judgment of the clinic physician. All invited individuals had CD4 counts below 350 cells/mm^3^ and had not previously received ART. As ART is provided exclusively through the China CARES system, misrepresentation of treatment history by subjects was considered unlikely.

Two hundred thirty-seven individuals were invited to participate; 20 declined. Plasma samples were obtained from the 217 consenting individuals before beginning ARV and stored at the Hunan Provincial Center for Disease Control facility. Of these, 90 samples were selected randomly to be examined for prevalence of WHO NRTI and NNRTI TDRs by deep sequencing.

This was a cross-sectional pilot prevalence study and the number of subjects with TDRs was not sufficient to examine the associations, if any, between TDR and demographic characteristics, risk categories for HIV infection, viral load at presentation, CD4 or other clinical data.

### CD4^+^ T cells absolute count and Viral load testing

CD4+ T cell count was measured by flow cytometer (FACS Calibur, BD Bioscience, USA). HIV-1 RNA viral load was quantified with the COBAS AmpliPrep COBAS TaqMan HIV-1 test (Roche Diagnostics Systems), version 2.0 (CAP/CTM v2.0) with a lower limit of detection of 40 RNA c/ml.

### Deep Sequencing

Samples from ART-naïve subjects were evaluated by deep sequencing [16], [21]–[25] for low-frequency HIV variants possessing WHO TDRs to 1% variant levels. Plasma samples were collected before starting therapy and stored in aliquots at −80°C until processed for deep sequencing. HIV-1 viral RNA was extracted from 140 ul of plasma samples using QIAmp RNA Mini Kit (Qiagen, Germany). The average plasma HIV viral load was 177,157 c/ml (median 73600 c/ml; range 3,980 to 1,560,000 c/ml). Deep sequencing was performed at Roche Application Support Center Laboratory in Shanghai, China. The extracted viral RNA was sent to the application support center (Roche Applied Science, Asia Pacific) for deep sequencing with 2 ug of the included polyA-RNA carrier.

Dried down proprietary sets of specific primers targeted to HIV *pol* were used to generate 4 overlapping amplicons at ∼400 bp (see Table S1 for details). cDNA synthesis and the amplicon generation was performed on 10 samples in a microtiter plate with specific primers targeted to four overlapping gene regions, encompassing about 1.3 kb sequence of the HIV *pol* region, include protease (PR) and reverse transcriptase (RT) genes. Four amplicons (∼400 bp in length) were amplified for each sample, and the fusion primers contained the Roche 454 amplicon adaptor sequences and multiplex identifier (MID) tags on both forward and reverse primers (see Table S1a for plate layout and Table S1b and Table S1c for primer sequences). Each amplicon was purified with Agencourt AMPure XP magnetic beads (Beckman Coulter, Beverly, MA, US) at a bead: DNA volume ratio of 0.8∶1, and quantified by PicoGreen Fluorescence using Quant-iT PicoGreen dsDNA Assay Kit(Invitrogen, Life Technologies, US). Agilent 2100bioanalyzer (Agilent Life Science, Santa Clara, California, US) was used to verify the quality and length of the amplicons. After quality controls, each set of 40 amplicons from a group of 10 samples were pooled, amplified by emPCR and sequenced in one GS Junior (454 Life Sciences, Roche, Branford, CT, US) run according to the manufacturer's sequencing protocol. Amplicons were pyrosequenced in both forward and reverse directions, with read depths >1000X (RT IQR 3,313–9,180 and PI IQR 3,356–9,152).

The sequence reads were analyzed with the GS Amplicon Variant Analyzer (AVA) software (Roche) which assigns each read to the proper amplicon and patient sample, using the multiplex identifier (MID) tags. AVA software was used for mapping and calculating variant frequencies at each nucleotide position relative to the sequence of HIV-1 reference strain HXB2 sequence and Drug Resistant Mutations (DRMs) were counted according to a predefined DRMs list [26]. Deep sequencing reproducibility and level of variant detection have been previously reported [11], [16], [18], [21]–[25], [28] to allow the detection of low-frequency variants' sensitivity down to approximately 0.5% variant detection level depending upon the sample HIV viral load (more than 10,000 c/ml). In this study we reported a variant detection limit of >1% as drug resistant variants at this level have been shown to be clinically relevant [16]–[20].

### Resistance Mutation Detection and Analysis

The full deep sequencing results are available through http://www.ncbi.nlm.nih.gov/sra; submission ID: Hunan CDC: 454 HIV Hunan China PRJNAA242262; sample numbers: SRS579392; SRS579686; SRS579687–SRS579774 (link to metadata: ftp://ftp-trace.ncbi.nlm.nih.gov/sra/review/SRP040396_20140325_173500_bca7ba75caac67b75fa8c9a101f25bf1).

All amino acid positions associated with antiretroviral resistance according to the 2009 edition of the WHO Transmitted Drug Resistance Mutations (TDRs) list [26] and Stanford HIV drug resistance database (hivdb.stanford.edu) were evaluated [27]. WHO list emphasizes mutations that are thought not to be polymorphic and therefore more likely to be TDRs. The Stanford resistance database includes all polymorphic mutations that may contribute to low levels of drug resistance. WHO TDRs for RT: 41L, K65R, D67N/G/E/del, T69D/Ins, K70R/E, L74V/I, V75M/T/A/S, F77L, Y115F, F116Y, Q151M, M184V/I, L210W, T215Y/F/I/S/C/D/V/E, K219Q/E/N/R [26].

Statistical analysis was performed using PASW Statistics version 13 (IBM SPSS, Chicago, Illinois). Any differences with p<0.05 were considered statistically significant and potentially relevant.

## Results

A total of 90 HIV-1 infected ART-naïve patients were selected in this study from July 2011 to June 2012, of which 20 were from Changsha and 70 from Hengyang. Sixty-five (72.2%) were male and 25 (27.7%) were female. The average age was 37 years (range: 20 to 70 years). Of the 90 subjects, HIV infection was diagnosed in 14.4% (13/90) in 2011, 62.2% (56/90) in 2010 and 23.3% (21/90) were confirmed before 2009. HIV transmission risks were heterosexual contact 62.2%, injection drug use 25.6%, male-to-male sexual contact 11.1%, and unreported 1.1%, see Table 1. Educational level was varied with 3 cases (3.33%) of illiteracy, 9 (10.0%) graduated from primary school, 42 (46.7%) from junior middle school, 27 (30.0%) from senior middle school or technical secondary school, and 9 (10.0%) from college or university.

**Table 1:** HIV subtype and HIV transmission risk category for 90 ART-naive subjects.

HIV Subtype HIV Risk Category	CRF01AE	B	C	G	Total
Heterosexual	46	5	3	1	55
MSM	6	3	1	0	10
IDU	21	0	1	0	22
IDU And Heterosexual	1	0	0	0	1
Blood	0	1	0	0	1
unrevealed	1	0	0	0	1
Total	75	9	5	1	90

### CD4 counts, HIV viral loads, and HIV subtype

The HIV viral loads for the 90 subjects ranged from 3,980 to 1,560,000 c/ml, with an average of 177,157 c/ml (median is 73,600 c/ml). The average CD4 cell count was 134 cells/mm^3^ (range 8 to 332 cells/mm^3^; median 101.5 cells/mm^3^). Eighty-three percent (75/90) of samples were AE subtype; 10% B, 5.6% C and 1 was subtype G; see Table 1 (HIV subtype based on hivdb.stanford.edu interpretation or HIV *pol* sequences).

### Deep Sequencing for HIV drug resistance mutation in RT

Overall 25.6% (23/90) of the subjects had HIV variants with a major WHO NRTI or NNRTI TDRs by deep sequencing at a variant frequency level >1% (range 1.02–16.67%), see Table 2. Fifteen subjects (16.7%) had HIV variants with NRTI TDRs; specific TDRs: M41L, K65R, D67G/N, L74V, F77L, M184I, T215I/S, and K219Q. Eleven subjects (12.2%) had HIV variants with a major NNRTI TDR: K101E, V106A, Y188C/H, G190E/A, and P225H. Specific NRTI and NNRTI mutations and HIV variant levels for each subject with a TDR are listed in Table 3. In addition, Sanger sequencing of RT was successful in 89/90 subjects. Three subjects had HIV with WHO TDR by Sanger sequencing (all with RT T69NT) that were missed by deep sequencing.

**Table 2:** Number of Subjects with a WHO TDR by Deep Sequencing*.

WHO TDR Class	
**NNRTI**	**11 (12.2%)**
**NRTI**	**15 (16.7%)**
**NRTI or NNRTI**	**23 (25.6%)**

*Number of subjects with a major WHO NRTI or NNRTI TDRs by deep sequencing at a HIV variant frequency level >1%. Percent (%) based on a total of 90 subjects with a deep sequencing result. WHO TDRs as listed by Bennett and colleagues (26).

**Table 3:** HIV drug resistance reverse transcriptase (RT) mutation detection by deep sequencing (DS).

No.	WHO RT TDR >1%	WHO TDR Class >1% Variant level	STANFORD RT TDR>1%	STANFORD HIVdb*
1001A	**M184I(1.36%)**	**NRTI**	**M184I(1.36%)**, V179D(1.22%)	**H-NRTI**
1014A	**V106A(4.94%)**	**NNRTI**	**V106A(4.94%)**	**H-NNRTI (NVP)**, I
1022A	M41L(2.41%)	NRTI	M41L(2.41%), V179D(85.25%)	S, PL
1051A	P255H(1.1%)	NNRTI	P255H(1.1%)	I, LL
3006A	**K65R(1.5%)**, T215I(2.04%)	**NRTI**	**K65R(1.5%)**,T69N(1.18%), V179I(2.71%), V179D(1.02%), T215I(2.04%)	**H-NRTI**, I
3013A	**V188C(1.02%)**	**NNRTI**	**V188C(1.02%)**	**H-NNRTI (NVP)**
3019A	P225H(1.32%)	NNRTI	P225H(1.32%)	I, LL
3021A	**M184I(1.17%)**	**NRTI**	V106I(2.38%), V118I(2.36%), V179I(5.73%), V179D(2.55%), **M184I(1.17%)**	**H-NRTI**, LL
3024A	D67G(1.79%), **G190E(3.36%)**	NRTI, **NNRTI**	D67G(1.79%), V75L(1.82%), V179I(2.41%), V179T(5.28%), V179D(3.72%), **G190E(3.36%)**, H221Y(1.59%)	**H-NNRTI (NVP)**
3029A	D67N(1.15%)	NRTI	D67N(1.15%)	LL
3058A	T215S(1.99%) K65N(8.13%)	NRTI	K65N(8.13%), V179D(25.77%),T215S(1.99%)	PL, LL
3061A	**Y188C(1.27%)**	**NNRTI**	T69N(1.67%), **Y188C(1.27%)**	**H-NNRTI (NVP)**
3079A	D67G(3.74%)	NRTI	D67E(1.53%), D67G(3.74%)	LL
3088A	K101E(1.42%)	NNRTI	K101E(1.42%), V179D(10.56%), F227L(1.35%)	I
3089A	K219Q(3.24%)	NRTI	K219Q(3.24%)	PL
3096A	D67G(1.03%)	NRTI	D67G(1.03%)	LL
3098A	P225H(1.69%)	NNRTI	T69N(66.55%),P225H(1.69%)	I, LL
3106A	**L74V(7.63%)**	**NRTI**	**L74V(7.63%)**	**H-NRTI, I**
3132A	**Y188H(1.63%)**	**NNRTI**	V179D(3.95%), **Y188H(1.63%)**	**H-NNRTI (NVP)**
3134A	D67G(3.04%), **Y188H(16.67%)**	NRTI, **NNRTI**	D67G(3.04%), V179D(7.75%), **Y188H(16.67%)**	**H-NNRTI (NVP)**
3138A	F77L(2.4%), **G190A(1.41%)**	NRTI, **NNRTI**	K70T(1.18%), F77L(2.4%), E138K(1.73%), V179T(6.1%), V179D(1.56%), **G190A(1.41%)**	**H-NNRTI (NVP)**
3146A	D67G(4.45%)	NRTI	D67G(4.45%)	LL
3157A	D67G(10.83%)	NRTI	D67G(10.83%), T69N(1.05%)	LL

*Stanford HIVdb algorithm interpretation abbreviations: H – High level resistance; I – Intermediate level resistance; LL – Low level resistance; PL – potential low level resistance. NVP - Nevirapine. High level resistance conferring mutations are in Bold. World Health Organization (WHO) Transmitted drug resistance mutation (TDR) [26]. TDR reported if the viral variant level >1% by deep sequencing.

Identified mutations were also interpreted using the HIVdb.stanford.edu algorithm to infer drug susceptibility and 18.9% of subjects (17/90) had viral variants with intermediate or high level resistance to NRTI or NNRTI when analyzed; 7.8% of subjects had viral variants at levels >1% with high-level resistance to nevirapine and 4.4% with high-level resistance to NRTIs (see Table 3).

To qualify for ART in 2010–2011 in this region a subject had to have a CD4 count <350 cells/mm^3^. Thus all patients deep sequenced had advanced disease and were likely chronically infected. Demographic, disease stage and virologic information for those with and without a WHO TDR is presented in Table 4. Given that this was a pilot study the data is presented descriptively as it was designed to determine prevalence of WHO TDRs and not to examine associations between groups.

**Table 4:** Baseline Characteristics of HIV-1 infected patients stratified by WHO RT TDR.

Characteristic	With WHO RT TDR *n* = 23 (%)	Without WHO RT TDR *n* = 67 (%)	Overall N = 90
Gender			
Male	17(73.9%)	48(71.6%)	65
Female	6(26.1%)	19(28.4%)	25
Marriage			
Married	12(52.2%)	32(47.8%)	34
Single	11(47.8%)	35(52.2%)	46
Age			
20∼29	4(17.4%)	18(26.9%)	22
30∼39	10(43.5%)	19(28.4%)	29
40∼49	5(21.7%)	22(32.8%)	27
50∼59	1(4.4%)	7(10.4%)	8
60∼	3(13.0%)	1(1.5%)	4
CD4 cell abs count(cells/mm3)			
0≤199	15(65.2%)	47(70.1%)	62
200∼349	8(34.8%)	20(29.9%)	28
≥350	0(0.0%)	0(0.0%)	0
HIV RNA median (copies/mL)	77100	73300	73600
Infection of the patients' spouse			
Negative	5(21.7%)	12(17.9%)	17
Positive	6(26.1%)	14(20.9%)	20
No spouse	8(34.8%)	25(37.3%)	33
Unrevealed	4(17.4%)	16(23.9%)	20
Route of transmission			
MSM	2(8.7%)	8(11.9%)	10
IDU	8(34.8%)	15(22.4%)	23
Heterosexual	13(56.5%)	42(62.7%)	55
IDU and heterosexual	0(0.0%)	1(1.5%)	1
Unrevealed	0(0.0%)	1(1.5%)	1
WHO staging	_________	_________	_________
I	12(52.2%)	48(71.6%)	60
II	1(4.3%)	3(4.5%)	4
III	10(43.5%)	15(22.4%)	25
IV	0(0.0%)	1(1.5%)	1

### Deep Sequencing for HIV PI drug resistance

Deep sequencing for PI mutations was successful with 87 of 90 (96.7%) unique ART-naïve patient samples −83% were subtype AE. Among 87 ART-naïve subjects, 24 (27.6%) had a viral variant with either a WHO TDR or Stanford.hivdb.edu PI mutation (algorithm value ≥15), Table 5. 17(19.5%) had WHO PI TDRs at levels >1%: V32I, M46I, I47V, G48V, I50V, F53L, I54T, Q58E, and V82T/A (variant levels range 1.04 to 4.6%). Stanford.hivdb.edu PI mutations were identified in samples from 21 subjects (24.1%) (variant range 1.1 to 99.8%); the most frequent and highest HIV variant level identified was T74S in 10 subjects (11.5%); T74S has a value of 15 only for nelfinavir. All low-level variants were interpreted by hivdb.stanford.edu, 8(9.2%) subjects had HIV variants with intermediate or high-level PI resistance (driven by V32I, I47V, G48V, I50V & V82A/T); 2 (2.3%) subjects had HIV variants with multiple PI mutations. Sanger sequencing identified 3 additional subjects had HIV with WHO PI TDRs (I50I/V, I54I/T, N88D/N) that were missed by deep sequencing.

**Table 5:** HIV drug resistance PI mutation detection by deep sequencing.

No.	WHO PI TDR>1%	STANFORD PI TDR>1%	Stanford Mutation* Level
1001A	none	T74S(2.23%)	L
1007A	none	T74S(11.66%)	L
1014A	none	T74S(1.66%)	L
1017A	none	T74S(2.41%)	L
1025A	V82T(1.64%)	V82T(1.64%),V82I((1)(93.96%),V82I(3)(2.08%)	I
1032A	V32I(4.6%)	V32I(4.6%),M46I(1.13%),T74S(4.73%)	I
1038A	I50V(1.87%)	I50V(1.87%)	H
1047A	Q58E(1)(2.64%)	Q58E(2.64%)	L
1048A	F53L(1.04%)	F53L(1.04%)	L
1055A	G48V(1.41%)	G48V(1.41%)	H
1064A	G48V(1.27%)	G48V(1.27%)	H
3009A	I47V(1.09%)	I47V(1.09%)	I
3012A	F53L(1.04%)	F53L(1.04%), T74S(40.56%)	L
3019A	I50V(1.41%)	I50V(1.41%), T74S(5%)	H
3025A	I54T(1.79%)	I54T(1.79%)	L
3039A	none	T74S(99.81%)	L
3042A	none	T74S(89.37%)	L
3058A	none	T74S(9.7%)	L
3074A	V82A(1.56%)	V82A(1.56%)	I
3078A	F53L(2.61%)	F53L(2.61%)	L
3088A	M46I(2.8%)	M46I(2.8%)	L
3128A	M46I(1.5%)	M46I(1.5%)	L
3132A	M46I(1.38%)	M46I(1.38%)	L
3134A	M46I(3.58%)	M46I(3.58%)	L

*Stanford HIVdb algorithm interpretation abbreviations: H – High level resistance; I – Intermediate level resistance; L – Low level resistance; PL – potential low level resistance. HIVdb.stanford.edu [27] PI scores accessed March 21, 2014. World Health Organization (WHO).

Overall 3 subjects had virus with dual class TDR at a HIV variant frequency level ≥1%. No subject had triple class resistance detected.

## Discussion

This study aimed to elucidate the prevalence of HIV with WHO TDRs by deep sequencing in ART-naïve subjects, since TDRs may affect the efficacy of the first line ART in Hunan Province, China. We identified that the predominant HIV subtype was CRF01_AE (83.3%, 75/90). This result is similar to that previously reported from Hunan Province molecular epidemiology HIV survey (2009–2012) which reported that 4 HIV-1 subtypes are circulating in Hunan (subtypes AE, BC, B and C) and that CRF_01AE was the dominant subtype representing more than 70% of the infections [1]–[4]; Hunan HIV epidemiology and treatment database system).

Among the 90 ART-naïve subjects evaluated in this study, 25.6% of subjects harbored HIV variants possessing WHO NRTI/NNRTI TDRs by deep sequencing that would affect the efficacy of the first line ART used in the region. The most frequently identified TDRs present at >1% levels were D67G/N, M184I, K65N/R, T215I/S, P225H, Y188C/H, K101E, V106A, and G190 A/E. All of the RT TDRs identified by deep sequencing were also found in another HIV drug resistance mutation study that used standard genotyping in Hunan province in 2009 [4]. Both these studies suggest that WHO TDR affecting first line therapy are circulating in Hunan Province.

The mutations identified in our study are ones that can be selected for by the ARTs in wide use in Hunan (d4T, ZDV, TDF, 3TC, and NVP). Of note is that the most common NNRTI mutations identified in this study are frequently selected for by NVP. The most common TDR mutation in Western countries (NNRTI – K103N mutation) where subtype B predominates was noticeably absent in our survey. The most common NNRTI TDR mutations identified in this study where >80% of the subjects are infected with subtype AE were: K101E, V106A, Y188C/H, and G190E/A. K101E and Y188C/H cause intermediate or high-level resistance to nevirapine (NVP) and low level resistance to efavirenz (EFV); V106A and G190A/E cause high level resistance to NVP and EFV [27]. We identified one subject with a L74V at a HIV variant level of 7.6%. The L74V RT mutation reduces susceptibility to ddI and ABC. ddI and ABC were not used in Hunan province, and the presence of this variant may represent a TDR acquired in another region or country. Sanger sequencing identified additional TDRs in a few subjects, however, different PCR primer sets were used for Sanger sequencing and the detection of additional mutations was likely due to the preferential amplification of different variants by the different primer sets.

Many of the mutations identified were at low levels <5%. It is likely that the majority of the subjects in this study had been infected for years as subjects had to have a CD4 counts below 350 cells/mm^3^ to receive ART through the national China CARES program during this time period. Given that these subjects were likely chronically infected (the majority had CD4 counts <200 cells/mm^3^) prior to presenting for ART initiation it may be that the mutant variants declined in frequency levels in relation to more fit wild type viruses over time. This has been noted in other studies [5], [17]–[19]. There were some subjects that had mutations at higher levels e.g. K65N, V179D/E, V106I, and Y188H.

Agents currently available for triple therapy in Hunan Province China are d4T, ZDV, TDF, 3TC, NVP, efavirenz (EFV), and lopinavir/ritonavir (LPV/r).

PI TDR mutations were identified frequently by deep sequencing (24% of subjects), but were found in isolation and at low variant frequency. The most frequent and highest HIV variant level identified was T74S in 10 subjects (11.5%); T74S has a value of 15 only for nelfinavir. All low-level variants were interpreted by Stanford.hivdb.edu, 9.2% of subjects had intermediate or high-level PI resistance (driven by V32I, I47V, G48V, I50V & V82A/T); only 2% of subjects had HIV variants with multiple PI mutations. As PI/r use is infrequent in Hunan, the existence of these PI mutations likely represent AE subtype natural polymorphism at low variant level frequency. Further deep sequencing studies are needed to better understand the prevalence of low-frequency variants possessing resistance mutations in AE subtype to better inform resistance algorithms and epidemiologic studies.

Our study has several limitations. First, in this pilot study we only evaluated 90 subjects by deep sequencing. This is a large number compared to other reports using deep sequencing, but the prevalence estimates would be better refined with a greater number of samples across multiple years. Second, we only reported low level variants to 1% levels. The 1% cut off was chosen because it is well established that low level variants >1% for NNRTI TDRs have clinical significance if a patient initiates a NNRTI based regimen [16]–[20]. Clonal mixing experiments were not performed as part of this study but there are reports that 454 deep sequencing can detect low level variants to 1% levels with good accuracy [11], [16], [18], [21]–[25], [28]–[35]. This level is above the known error rates for PCR and pyrosequencing, although errors occurring in early rounds of amplification can still affect the results; and the 1% level has been reported in prior TDR prevalence studies using deep sequencing [11], [16], [18], [21]–[25], [28]. Lastly, we cannot correlate the low level variant detection with clinical outcome as that was not the aim of this pilot study. In the future, we plan to investigate the effect of low level variants possessing TDR specifically in subtype AE on virologic response rates with the first line ART used in China.

## Conclusions

Using deep sequencing, we found that ART-naïve subjects from Hunan Province, China, who were infected predominantly with subtype AE, frequently possessed HIV variants with WHO NRTI/NNRTI/PI TDRs that would affect the first line ART used in the region. Specific mutations conferring high-level resistance to nevirapine were identified in 7.8% of the subjects. Many of the TDRs identified were at low variant levels (<5%) and may have been due to subjects having advanced chronic disease when tested (the majority had CD4 counts <200 cells/mm^3^ when presenting to initiate ART). This delay may have allowed time for mutant variants with TDRs to decline in frequency levels in relation to more fit wild type viruses. Ongoing surveillance is needed to monitor for TDRs in newly and chronically infected subjects in Hunan Province, China.

## Supporting Information

Table S1 Table S1a. Plate Layout for HIV deep sequencing. Table S1b. Forward primer sequences. Table S1c. Reverse primer sequences.
(DOCX)
