# Supplementary material for: Prevalence of WHO Transmitted Drug Resistance Mutations by Deep Sequencing in Antiretroviral-Naïve Subjects in Hunan Province, China
Source: PLoS One. 2014 Jun 4;9(6):e98740. doi: 10.1371/journal.pone.0098740 (PMC4045886; doi:10.1371/journal.pone.0098740)
Supplement: Table S1 — a. Plate Layout for HIV deep sequencing. Table S1b. Forward primer sequences. Table S1c. Reverse primer sequences. (DOCX) [file pone.0098740.s001.docx]

**Table Supplement 1a. Plate Layout for HIV deep sequencing**

| **NB. Each well of the plate contains one forward and one reverse primer** | | | | | | |  |  |  |  |  |  |
| --- | --- | --- | --- | --- | --- | --- | --- | --- | --- | --- | --- | --- |
| **Eg. RTP 1 with MID4 = fr primer for A1 and Rev primer for A1** | | | | | |  |  |  |  |  |  |  |
| **Eg. RTP 2 with MID4 = fr primer for B1 and Rev primer for B1** | | | | | |  |  |  |  |  |  |  |
|  |  |  |  |  |  |  |  |  |  |  |  |  |
|  | MID4 | MID20 | MID22 | MID23 | MID24 | MID25 | MID26 | MID27 | MID28 | MID29 | MID30 | noAdaptor |
|  | 1 | 2 | 3 | 4 | 5 | 6 | 7 | 8 | 9 | 10 | 11 | 12 |
| A | RTP I | RTP I | RTP I | RTP I | RTP I | RTP I | RTP I | RTP I | RTP I | RTP I | RTP I | RTP I |
| B | RTP II | RTP II | RTP II | RTP II | RTP II | RTP II | RTP II | RTP II | RTP II | RTP II | RTP II | RTP II |
| C | RTP III | RTP III | RTP III | RTP III | RTP III | RTP III | RTP III | RTP III | RTP III | RTP III | RTP III | RTP III |
| D | RTP IV | RTP IV | RTP IV | RTP IV | RTP IV | RTP IV | RTP IV | RTP IV | RTP IV | RTP IV | RTP IV | RTP IV |
|  |  |  |  |  |  |  |  |  |  |  |  |  |
|  |  |  |  |  |  |  |  |  |  |  |  |  |
|  |  |  |  |  |  |  |  |  |  |  |  |  |
|  |  |  |  |  |  |  |  |  |  |  |  |  |
|  |  |  |  |  |  |  |  |  |  |  |  |  |
|  | RTP 1F Multi + RTP 1R = RTP I | | |  |  |  |  |  |  |  |  |  |
|  | RTP 2F Multi + RTP 2R = RTP II | | |  |  |  |  |  |  |  |  |  |
|  | RTP 5F Multi + RTP 5R = RTP III | | |  |  |  |  |  |  |  |  |  |
|  | RTP 6F Multi + RTP 6R = RTP IV | | |  |  |  |  |  |  |  |  |  |
|  |  |  |  |  |  |  |  |  |  |  |  |  |

|  | **Table Supplement 1b. Forward primer sequences** |  |  |  | length |
| --- | --- | --- | --- | --- | --- |
| A1 | RTP 1F Multi MID4 | CGTATCGCCTCCCTCGCGCCATCAGAGCACTGTAGATCACTCTTTGGCARCGACC | |  | 55 |
| B1 | RTP 2F Multi MID4 | CGTATCGCCTCCCTCGCGCCATCAGAGCACTGTAGGGAATTGGAGGTTTTATCAARGT | |  | 58 |
| C1 | RTP 5F Multi MID4 | CGTATCGCCTCCCTCGCGCCATCAGAGCACTGTAGGTACCAGTAAAATTAAAGCCAGGRA | |  | 60 |
| D1 | RTP 6F Multi MID4 | CGTATCGCCTCCCTCGCGCCATCAGAGCACTGTAGCACCAGGGATTAGATATCAGTACAATGT | |  | 63 |
| E1 | Empty |  |  |  |  |
| F1 | Empty |  |  |  |  |
| G1 | Empty |  |  |  |  |
| H1 | Empty |  |  |  |  |
| A2 | RTP 1F Multi MID20 | CGTATCGCCTCCCTCGCGCCATCAGACGACTACAGATCACTCTTTGGCARCGACC | |  | 55 |
| B2 | RTP 2F Multi MID20 | CGTATCGCCTCCCTCGCGCCATCAGACGACTACAGGGAATTGGAGGTTTTATCAARGT | |  | 58 |
| C2 | RTP 5F Multi MID20 | CGTATCGCCTCCCTCGCGCCATCAGACGACTACAGGTACCAGTAAAATTAAAGCCAGGRA | |  | 60 |
| D2 | RTP 6F Multi MID20 | CGTATCGCCTCCCTCGCGCCATCAGACGACTACAGCACCAGGGATTAGATATCAGTACAATGT | |  | 63 |
| E2 | Empty |  |  |  |  |
| F2 | Empty |  |  |  |  |
| G2 | Empty |  |  |  |  |
| H2 | Empty |  |  |  |  |
| A3 | RTP 1F Multi MID22 | CGTATCGCCTCCCTCGCGCCATCAGTACGAGTATGATCACTCTTTGGCARCGACC | |  | 55 |
| B3 | RTP 2F Multi MID22 | CGTATCGCCTCCCTCGCGCCATCAGTACGAGTATGGGAATTGGAGGTTTTATCAARGT | |  | 58 |
| C3 | RTP 5F Multi MID22 | CGTATCGCCTCCCTCGCGCCATCAGTACGAGTATGGTACCAGTAAAATTAAAGCCAGGRA | |  | 60 |
| D3 | RTP 6F Multi MID22 | CGTATCGCCTCCCTCGCGCCATCAGTACGAGTATGCACCAGGGATTAGATATCAGTACAATGT | |  | 63 |
| E3 | Empty |  |  |  |  |
| F3 | Empty |  |  |  |  |
| G3 | Empty |  |  |  |  |
| H3 | Empty |  |  |  |  |
| A4 | RTP 1F Multi MID23 | CGTATCGCCTCCCTCGCGCCATCAGTACTCTCGTGATCACTCTTTGGCARCGACC | |  | 55 |
| B4 | RTP 2F Multi MID23 | CGTATCGCCTCCCTCGCGCCATCAGTACTCTCGTGGGAATTGGAGGTTTTATCAARGT | |  | 58 |
| C4 | RTP 5F Multi MID23 | CGTATCGCCTCCCTCGCGCCATCAGTACTCTCGTGGTACCAGTAAAATTAAAGCCAGGRA | |  | 60 |
| D4 | RTP 6F Multi MID23 | CGTATCGCCTCCCTCGCGCCATCAGTACTCTCGTGCACCAGGGATTAGATATCAGTACAATGT | |  | 63 |
| E4 | Empty |  |  |  |  |
| F4 | Empty |  |  |  |  |
| G4 | Empty |  |  |  |  |
| H4 | Empty |  |  |  |  |
| A5 | RTP 1F Multi MID24 | CGTATCGCCTCCCTCGCGCCATCAGTAGAGACGAGATCACTCTTTGGCARCGACC | |  | 55 |
| B5 | RTP 2F Multi MID24 | CGTATCGCCTCCCTCGCGCCATCAGTAGAGACGAGGGAATTGGAGGTTTTATCAARGT | |  | 58 |
| C5 | RTP 5F Multi MID24 | CGTATCGCCTCCCTCGCGCCATCAGTAGAGACGAGGTACCAGTAAAATTAAAGCCAGGRA | |  | 60 |
| D5 | RTP 6F Multi MID24 | CGTATCGCCTCCCTCGCGCCATCAGTAGAGACGAGCACCAGGGATTAGATATCAGTACAATGT | | | 63 |
| E5 | Empty |  |  |  |  |
| F5 | Empty |  |  |  |  |
| G5 | Empty |  |  |  |  |
| H5 | Empty |  |  |  |  |
| A6 | RTP 1F Multi MID25 | CGTATCGCCTCCCTCGCGCCATCAGTCGTCGCTCGATCACTCTTTGGCARCGACC | |  | 55 |
| B6 | RTP 2F Multi MID25 | CGTATCGCCTCCCTCGCGCCATCAGTCGTCGCTCGGGAATTGGAGGTTTTATCAARGT | |  | 58 |
| C6 | RTP 5F Multi MID25 | CGTATCGCCTCCCTCGCGCCATCAGTCGTCGCTCGGTACCAGTAAAATTAAAGCCAGGRA | |  | 60 |
| D6 | RTP 6F Multi MID25 | CGTATCGCCTCCCTCGCGCCATCAGTCGTCGCTCGCACCAGGGATTAGATATCAGTACAATGT | |  | 63 |
| E6 | Empty |  |  |  |  |
| F6 | Empty |  |  |  |  |
| G6 | Empty |  |  |  |  |
| H6 | Empty |  |  |  |  |
| A7 | RTP 1F Multi MID26 | CGTATCGCCTCCCTCGCGCCATCAGACATACGCGTATCACTCTTTGGCARCGACC | |  | 55 |
| B7 | RTP 2F Multi MID26 | CGTATCGCCTCCCTCGCGCCATCAGACATACGCGTGGAATTGGAGGTTTTATCAARGT | |  | 58 |
| C7 | RTP 5F Multi MID26 | CGTATCGCCTCCCTCGCGCCATCAGACATACGCGTGTACCAGTAAAATTAAAGCCAGGRA | |  | 60 |
| D7 | RTP 6F Multi MID26 | CGTATCGCCTCCCTCGCGCCATCAGACATACGCGTCACCAGGGATTAGATATCAGTACAATGT | |  | 63 |
| E7 | Empty |  |  |  |  |
| F7 | Empty |  |  |  |  |
| G7 | Empty |  |  |  |  |
| H7 | Empty |  |  |  |  |
| A8 | RTP 1F Multi MID27 | CGTATCGCCTCCCTCGCGCCATCAGACGCGAGTATATCACTCTTTGGCARCGACC | |  | 55 |
| B8 | RTP 2F Multi MID27 | CGTATCGCCTCCCTCGCGCCATCAGACGCGAGTATGGAATTGGAGGTTTTATCAARGT | |  | 58 |
| C8 | RTP 5F Multi MID27 | CGTATCGCCTCCCTCGCGCCATCAGACGCGAGTATGTACCAGTAAAATTAAAGCCAGGRA | |  | 60 |
| D8 | RTP 6F Multi MID27 | CGTATCGCCTCCCTCGCGCCATCAGACGCGAGTATCACCAGGGATTAGATATCAGTACAATGT | |  | 63 |
| E8 | Empty |  |  |  |  |
| F8 | Empty |  |  |  |  |
| G8 | Empty |  |  |  |  |
| H8 | Empty |  |  |  |  |
| A9 | RTP 1F Multi MID28 | CGTATCGCCTCCCTCGCGCCATCAGACTACTATGTATCACTCTTTGGCARCGACC | |  | 55 |
| B9 | RTP 2F Multi MID28 | CGTATCGCCTCCCTCGCGCCATCAGACTACTATGTGGAATTGGAGGTTTTATCAARGT | |  | 58 |
| C9 | RTP 5F Multi MID28 | CGTATCGCCTCCCTCGCGCCATCAGACTACTATGTGTACCAGTAAAATTAAAGCCAGGRA | |  | 60 |
| D9 | RTP 6F Multi MID28 | CGTATCGCCTCCCTCGCGCCATCAGACTACTATGTCACCAGGGATTAGATATCAGTACAATGT | |  | 63 |
| E9 | Empty |  |  |  |  |
| F9 | Empty |  |  |  |  |
| G9 | Empty |  |  |  |  |
| H9 | Empty |  |  |  |  |
| A10 | RTP 1F Multi MID29 | CGTATCGCCTCCCTCGCGCCATCAGACTGTACAGTATCACTCTTTGGCARCGACC | |  | 55 |
| B10 | RTP 2F Multi MID29 | CGTATCGCCTCCCTCGCGCCATCAGACTGTACAGTGGAATTGGAGGTTTTATCAARGT | |  | 58 |
| C10 | RTP 5F Multi MID29 | CGTATCGCCTCCCTCGCGCCATCAGACTGTACAGTGTACCAGTAAAATTAAAGCCAGGRA | |  | 60 |
| D10 | RTP 6F Multi MID29 | CGTATCGCCTCCCTCGCGCCATCAGACTGTACAGTCACCAGGGATTAGATATCAGTACAATGT | |  | 63 |
| E10 | Empty |  |  |  |  |
| F10 | Empty |  |  |  |  |
| G10 | Empty |  |  |  |  |
| H10 | Empty |  |  |  |  |
| A11 | RTP 1F Multi MID30 | CGTATCGCCTCCCTCGCGCCATCAGAGACTATACTATCACTCTTTGGCARCGACC | |  | 55 |
| B11 | RTP 2F Multi MID30 | CGTATCGCCTCCCTCGCGCCATCAGAGACTATACTGGAATTGGAGGTTTTATCAARGT | |  | 58 |
| C11 | RTP 5F Multi MID30 | CGTATCGCCTCCCTCGCGCCATCAGAGACTATACTGTACCAGTAAAATTAAAGCCAGGRA | |  | 60 |
| D11 | RTP 6F Multi MID30 | CGTATCGCCTCCCTCGCGCCATCAGAGACTATACTCACCAGGGATTAGATATCAGTACAATGT | |  | 63 |
| E11 | Empty |  |  |  |  |
| F11 | Empty |  |  |  |  |
| G11 | Empty |  |  |  |  |
| H11 | Empty |  |  |  |  |
| A12 | RTP 1F Multi noAdaptor | ATCACTCTTTGGCARCGACC | |  | 20 |
| B12 | RTP 2F Multi noAdaptor | GGAATTGGAGGTTTTATCAARGT | |  | 23 |
| C12 | RTP 5F Multi noAdaptor | GTACCAGTAAAATTAAAGCCAGGRA | |  | 25 |
| D12 | RTP 6F Multi noAdaptor | CACCAGGGATTAGATATCAGTACAATGT | |  | 28 |
| E12 | Empty |  |  |  |  |
| F12 | Empty |  |  |  |  |
| G12 | Empty |  |  |  |  |
| H12 | Empty |  |  |  |  |

|  | **Table Supplement 1c. Reverse primer sequences** |  | length |
| --- | --- | --- | --- |
| A1 | *RTP 1R Multi MID4* | CTATGCGCCTTGCCAGCCCGCTCAGAGCACTGTAGTTGGGCCATCCATTCCTGG | 54 |
| B1 | *RTP 2R Multi MID4* | CTATGCGCCTTGCCAGCCCGCTCAGAGCACTGTAGTGTGGTATTCCTAATTGAACYTCCCA | 61 |
| C1 | *RTP 5R Multi MID4* | CTATGCGCCTTGCCAGCCCGCTCAGAGCACTGTAGGGCTCTAAGATTTTTGTCATGCT | 58 |
| D1 | *RTP 6R Multi MID4* | CTATGCGCCTTGCCAGCCCGCTCAGAGCACTGTAGAACTTCTGTATATCATTGACAGTCCA | 61 |
| E1 | *Empty* |  |  |
| F1 | *Empty* |  |  |
| G1 | *Empty* |  |  |
| H1 | *Empty* |  |  |
| A2 | *RTP 1R Multi MID20* | CTATGCGCCTTGCCAGCCCGCTCAGACGACTACAGTTGGGCCATCCATTCCTGG | 54 |
| B2 | *RTP 2R Multi MID20* | CTATGCGCCTTGCCAGCCCGCTCAGACGACTACAGTGTGGTATTCCTAATTGAACYTCCCA | 61 |
| C2 | *RTP 5R Multi MID20* | CTATGCGCCTTGCCAGCCCGCTCAGACGACTACAGGGCTCTAAGATTTTTGTCATGCT | 58 |
| D2 | *RTP 6R Multi MID20* | CTATGCGCCTTGCCAGCCCGCTCAGACGACTACAGAACTTCTGTATATCATTGACAGTCCA | 61 |
| E2 | *Empty* |  |  |
| F2 | *Empty* |  |  |
| G2 | *Empty* |  |  |
| H2 | *Empty* |  |  |
| A3 | *RTP 1R Multi MID22* | CTATGCGCCTTGCCAGCCCGCTCAGTACGAGTATGTTGGGCCATCCATTCCTGG | 54 |
| B3 | *RTP 2R Multi MID22* | CTATGCGCCTTGCCAGCCCGCTCAGTACGAGTATGTGTGGTATTCCTAATTGAACYTCCCA | 61 |
| C3 | *RTP 5R Multi MID22* | CTATGCGCCTTGCCAGCCCGCTCAGTACGAGTATGGGCTCTAAGATTTTTGTCATGCT | 58 |
| D3 | *RTP 6R Multi MID22* | CTATGCGCCTTGCCAGCCCGCTCAGTACGAGTATGAACTTCTGTATATCATTGACAGTCCA | 61 |
| E3 | *Empty* |  |  |
| F3 | *Empty* |  |  |
| G3 | *Empty* |  |  |
| H3 | *Empty* |  |  |
| A4 | *RTP 1R Multi MID23* | CTATGCGCCTTGCCAGCCCGCTCAGTACTCTCGTGTTGGGCCATCCATTCCTGG | 54 |
| B4 | *RTP 2R Multi MID23* | CTATGCGCCTTGCCAGCCCGCTCAGTACTCTCGTGTGTGGTATTCCTAATTGAACYTCCCA | 61 |
| C4 | *RTP 5R Multi MID23* | CTATGCGCCTTGCCAGCCCGCTCAGTACTCTCGTGGGCTCTAAGATTTTTGTCATGCT | 58 |
| D4 | *RTP 6R Multi MID23* | CTATGCGCCTTGCCAGCCCGCTCAGTACTCTCGTGAACTTCTGTATATCATTGACAGTCCA | 61 |
| E4 | *Empty* |  |  |
| F4 | *Empty* |  |  |
| G4 | *Empty* |  |  |
| H4 | *Empty* |  |  |
| A5 | *RTP 1R Multi MID24* | CTATGCGCCTTGCCAGCCCGCTCAGTAGAGACGAGTTGGGCCATCCATTCCTGG | 54 |
| B5 | *RTP 2R Multi MID24* | CTATGCGCCTTGCCAGCCCGCTCAGTAGAGACGAGTGTGGTATTCCTAATTGAACYTCCCA | 61 |
| C5 | *RTP 5R Multi MID24* | CTATGCGCCTTGCCAGCCCGCTCAGTAGAGACGAGGGCTCTAAGATTTTTGTCATGCT | 58 |
| D5 | *RTP 6R Multi MID24* | CTATGCGCCTTGCCAGCCCGCTCAGTAGAGACGAGAACTTCTGTATATCATTGACAGTCCA | 61 |
| E5 | *Empty* |  |  |
| F5 | *Empty* |  |  |
| G5 | *Empty* |  |  |
| H5 | *Empty* |  |  |
| A6 | *RTP 1R Multi MID25* | CTATGCGCCTTGCCAGCCCGCTCAGTCGTCGCTCGTTGGGCCATCCATTCCTGG | 54 |
| B6 | *RTP 2R Multi MID25* | CTATGCGCCTTGCCAGCCCGCTCAGTCGTCGCTCGTGTGGTATTCCTAATTGAACYTCCCA | 61 |
| C6 | *RTP 5R Multi MID25* | CTATGCGCCTTGCCAGCCCGCTCAGTCGTCGCTCGGGCTCTAAGATTTTTGTCATGCT | 58 |
| D6 | *RTP 6R Multi MID25* | CTATGCGCCTTGCCAGCCCGCTCAGTCGTCGCTCGAACTTCTGTATATCATTGACAGTCCA | 61 |
| E6 | *Empty* |  |  |
| F6 | *Empty* |  |  |
| G6 | *Empty* |  |  |
| H6 | *Empty* |  |  |
| A7 | *RTP 1R Multi MID26* | CTATGCGCCTTGCCAGCCCGCTCAGACATACGCGTTTGGGCCATCCATTCCTGG | 54 |
| B7 | *RTP 2R Multi MID26* | CTATGCGCCTTGCCAGCCCGCTCAGACATACGCGTTGTGGTATTCCTAATTGAACYTCCCA | 61 |
| C7 | *RTP 5R Multi MID26* | CTATGCGCCTTGCCAGCCCGCTCAGACATACGCGTGGCTCTAAGATTTTTGTCATGCT | 58 |
| D7 | *RTP 6R Multi MID26* | CTATGCGCCTTGCCAGCCCGCTCAGACATACGCGTAACTTCTGTATATCATTGACAGTCCA | 61 |
| E7 | *Empty* |  |  |
| F7 | *Empty* |  |  |
| G7 | *Empty* |  |  |
| H7 | *Empty* |  |  |
| A8 | *RTP 1R Multi MID27* | CTATGCGCCTTGCCAGCCCGCTCAGACGCGAGTATTTGGGCCATCCATTCCTGG | 54 |
| B8 | *RTP 2R Multi MID27* | CTATGCGCCTTGCCAGCCCGCTCAGACGCGAGTATTGTGGTATTCCTAATTGAACYTCCCA | 61 |
| C8 | *RTP 5R Multi MID27* | CTATGCGCCTTGCCAGCCCGCTCAGACGCGAGTATGGCTCTAAGATTTTTGTCATGCT | 58 |
| D8 | *RTP 6R Multi MID27* | CTATGCGCCTTGCCAGCCCGCTCAGACGCGAGTATAACTTCTGTATATCATTGACAGTCCA | 61 |
| E8 | *Empty* |  |  |
| F8 | *Empty* |  |  |
| G8 | *Empty* |  |  |
| H8 | *Empty* |  |  |
| A9 | *RTP 1R Multi MID28* | CTATGCGCCTTGCCAGCCCGCTCAGACTACTATGTTTGGGCCATCCATTCCTGG | 54 |
| B9 | *RTP 2R Multi MID28* | CTATGCGCCTTGCCAGCCCGCTCAGACTACTATGTTGTGGTATTCCTAATTGAACYTCCCA | 61 |
| C9 | *RTP 5R Multi MID28* | CTATGCGCCTTGCCAGCCCGCTCAGACTACTATGTGGCTCTAAGATTTTTGTCATGCT | 58 |
| D9 | *RTP 6R Multi MID28* | CTATGCGCCTTGCCAGCCCGCTCAGACTACTATGTAACTTCTGTATATCATTGACAGTCCA | 61 |
| E9 | *Empty* |  |  |
| F9 | *Empty* |  |  |
| G9 | *Empty* |  |  |
| H9 | *Empty* |  |  |
| A10 | *RTP 1R Multi MID29* | CTATGCGCCTTGCCAGCCCGCTCAGACTGTACAGTTTGGGCCATCCATTCCTGG | 54 |
| B10 | *RTP 2R Multi MID29* | CTATGCGCCTTGCCAGCCCGCTCAGACTGTACAGTTGTGGTATTCCTAATTGAACYTCCCA | 61 |
| C10 | *RTP 5R Multi MID29* | CTATGCGCCTTGCCAGCCCGCTCAGACTGTACAGTGGCTCTAAGATTTTTGTCATGCT | 58 |
| D10 | *RTP 6R Multi MID29* | CTATGCGCCTTGCCAGCCCGCTCAGACTGTACAGTAACTTCTGTATATCATTGACAGTCCA | 61 |
| E10 | *Empty* |  |  |
| F10 | *Empty* |  |  |
| G10 | *Empty* |  |  |
| H10 | *Empty* |  |  |
| A11 | *RTP 1R Multi MID30* | CTATGCGCCTTGCCAGCCCGCTCAGAGACTATACTTTGGGCCATCCATTCCTGG | 54 |
| B11 | *RTP 2R Multi MID30* | CTATGCGCCTTGCCAGCCCGCTCAGAGACTATACTTGTGGTATTCCTAATTGAACYTCCCA | 61 |
| C11 | *RTP 5R Multi MID30* | CTATGCGCCTTGCCAGCCCGCTCAGAGACTATACTGGCTCTAAGATTTTTGTCATGCT | 58 |
| D11 | *RTP 6R Multi MID30* | CTATGCGCCTTGCCAGCCCGCTCAGAGACTATACTAACTTCTGTATATCATTGACAGTCCA | 61 |
| E11 | *Empty* |  |  |
| F11 | *Empty* |  |  |
| G11 | *Empty* |  |  |
| H11 | *Empty* |  |  |
| A12 | *RTP 1R Multi noAdaptor* | TTGGGCCATCCATTCCTGG | 19 |
| B12 | *RTP 2R Multi noAdaptor* | TGTGGTATTCCTAATTGAACYTCCCA | 26 |
| C12 | *RTP 5R Multi noAdaptor* | GGCTCTAAGATTTTTGTCATGCT | 23 |
| D12 | *RTP 6R Multi noAdaptor* | AACTTCTGTATATCATTGACAGTCCA | 26 |
| E12 | *Empty* |  |  |
| F12 | *Empty* |  |  |
| G12 | *Empty* |  |  |
| H12 | *Empty* |  |  |
